# Measuring More Than Exposure: Does Stress Appraisal Matter for Black–White Differences in Anxiety and Depressive Symptoms Among Older Adults?

**DOI:** 10.1093/geroni/igaa040

**Published:** 2020-09-07

**Authors:** Lauren L Brown, Leah R Abrams, Uchechi A Mitchell, Jennifer A Ailshire

**Affiliations:** 1 Division of Health Management and Policy, San Diego State University School of Public Health, California; 2 Harvard Center for Population and Development Studies, Harvard T.H. Chan School of Public Health, Cambridge, Massachusetts; 3 Division of Community Health Sciences, School of Public Health, University of Illinois Chicago; 4 Leonard Davis School of Gerontology, University of Southern California, Los Angeles

**Keywords:** Aging, Chronic stress, Health and Retirement Study, Health disparities, Mental health

## Abstract

**Background and Objectives:**

Prior research and theory suggest that exposure to objectively stressful events contributes to mental health disparities. Yet, blacks report higher cumulative stress exposure than whites but lower levels of common psychiatric disorders. In order to understand why blacks bear disproportionate stress exposure but similar or better mental health relative to whites, we need to consider race differences in not only stress exposure, but also stress appraisal—how upsetting stress exposures are perceived to be.

**Research Design and Methods:**

We examine whether race differences in the number of reported chronic stressors across 5 domains (health, financial, residential, relationship, and caregiving) and their appraised stressfulness explain black–white differences in anxiety and depressive symptoms. Data come from 6019 adults aged older than 52 from the 2006 Health and Retirement Study.

**Results:**

Older blacks in this sample experience greater exposure to chronic stressors but appraise stressors as less upsetting relative to whites. In fully adjusted models, stress exposure is related to higher levels of anxiety and depressive symptoms, and perceiving stress as upsetting is associated with higher symptomology for whites and blacks. We also find that blacks report greater anxiety symptoms but fewer depressive symptoms with more stress exposure relative to whites. Stress appraisal partially explains race differences in the association between stress exposure and anxiety symptoms and fully explains race differences in the association between exposure and depressive symptoms.

**Discussion and Implications:**

The relationship between race, chronic stress exposure, and mental health is mediated by stress appraisal. Stress appraisal provides insight on important pathways contributing to black–white mental health disparities in older adulthood.

**Translational Significance:** Older blacks report greater exposure to chronic stressors across health, housing, relationship, financial, and caregiving domains that undoubtedly have consequences for their mental health and well-being. Yet, older blacks also appraise chronic stress exposure as less upsetting relative to whites, suggesting they respond to stress differently, adapting to differential historical and current lived experiences. Incorporating stress appraisal into disparities research may help us understand the similar or lower rates of depressive and anxiety disorders among blacks relative to non-Hispanic whites despite large disparities in stress exposure and physical health in mid- and late life—commonly referred to as the black–white mental health paradox. Practice and policy implications of the study call for connecting black older adults facing health, financial, and housing adversity to services that minimize mental-health–related repercussions. Alternatively, establishing financial and housing security as fundamental rights for older adults, especially black older adults, lessens exposure to chronic stressors that have detrimental mental health consequences.

Stress theory positions stress exposure as foundational in generating racial inequities in health. The *stress process model* and *differential exposure hypothesis* dominate this literature, suggesting that racial disparities in mental well-being are largely due to systematic and differential exposure to health-degrading social and economic stressors (1–6). National surveys have embedded differential exposure theories into how they measure stress among populations. While these operationalizations of stress have helped to establish stress exposure as a mechanism of racial differences in mental health (6–8), they overlook an important, subjective element of stress—stress appraisal. Psychological models of stress emphasize that experiencing the same event can be stressful for some individuals but not others—with how exactly an individual evaluates an ostensibly stressful event depending on one’s personal and social history, culture, and the context in which the event occurs (9–11). Simply counting up stress exposures, in the way national surveys propose, overlooks the subjective dimensions of the stress process and, thus, may not provide a complete picture of how stress affects mental health or generates racial health disparities.

Incorporating appraisal into stress measurement may be particularly important in understanding black–white patterning of mental health. In epidemiological research, black–white patterning in mental health varies by how mental health is measured. Most studies find that blacks have higher levels of psychological distress (12,13) compared to whites, but have a lower risk of most common psychiatric disorders (12,14). For instance, there is evidence of lower rates of depressive and anxiety disorders among blacks relative to non-Hispanic whites despite large disparities in stress exposure and physical health in mid- and late life—commonly referred to as the black–white mental health paradox (14–16). There is some evidence to suggest that the black–white paradox in mental health is an artifact of measurement error, implying existing measures do not effectively capture the psychopathology of blacks as well as they do for whites (17–20). However, there is limited support that the modest measurement inconsistencies that do exist for blacks and whites are sufficient in explaining the black–white mental health paradox (15,16).

Building on the idea that the stress experience is different for older blacks (21), we hypothesize that aspects of the stress experience are not captured or measured in population surveys that may inform our understanding of black–white patterning in mental health. This article will examine black–white differences in chronic stress exposure and appraisal as predictors of anxiety and depressive symptoms in older adulthood. Importantly, we use a measure of chronic stress that incorporates more facets of the stress process, specifically, the exposure and appraisal of chronic stressors across five life domains—health, financial, residential, relationship, and caregiving strain—to determine their impact on black and white older adults’ mental health.

## Background and Objectives

### The Black–White Paradox: Making a Case for Stress Appraisal

The black–white paradox in mental health suggests that unmeasured factors, outside of the traditional stress process framework, affect the relationship between race, stress, and mental health. Stress exposure is only one feature of the stress experience. For example, an older adult experiencing financial hardship while also caregiving for a spouse, theoretically, would endorse these exposures as stressful, referring to an imbalance between the demands of a stressor and the internal resources an individual has to cope with the stressor. Stress, therefore, has at least two measurable components: (a) exposure to the stressor and (b) the corresponding appraisal which is an evaluation of the perceived demands of the stressor and the resources or ability to cope (1,22). The differential vulnerability hypothesis constructs a theoretical rationale to incorporate the appraisal process into the health disparities literature. It suggests that, when levels of stress exposure are equal, socially disadvantaged groups and blacks, in particular, react more strongly to stressors because they have fewer social and personal resources to buffer the negative effects of stress on health (2,3). Racial minorities—with the dual burden of socioeconomic disadvantage and race-related stressors—may be at even greater risk because they have limited access to psychosocial and material coping mechanisms (23). Generally, studies examining race differences in exposure and vulnerability to stressful life events have found both greater exposure and psychological distress among low-socioeconomic status (SES) nonwhites (11,12,24). These theories and prior studies suggest blacks are disproportionately exposed to various social stressors, experience more distress, and should also position blacks to be at higher risk of poor mental health.

However, minority status, despite being related to experiences of prejudice, discrimination, greater stress exposure, and lower SES, is also a source of psychosocial resources, such as a collective racial identity (25), mastery (26,27), and larger and more supportive religious and social networks (28–30) that can protect against the effects of chronic stressors (31). It may be that racial/ethnic minorities are more prone to stress exposure, have less access to resources related to SES, but have adapted better coping mechanisms, and have access to other psychosocial resources that leave them better able to manage the mental health consequences of adversity relative to their white peers. Prior empirical evidence has shown that older blacks appraise stress to be less upsetting and report lower general and domain-specific appraisal of chronic stressors compared to whites (21), perhaps because these protective resources alter the stressfulness of a given exposure. Take mastery for example, mastery results from past experience, mostly. Mastery affects the degree to which stressors are perceived as problematic. Previously dealing with a stressor may increase the threshold that would accompany the first exposure to that same stressor (32). Older blacks have faced a lifetime of overt discrimination and race-related stressors—having come of age during Jim Crow, desegregation, the Civil Rights Movement, and now the Movement for black Lives that is responding to the disproportionate killing of black people by the police (33)—in addition to more mundane chronic strains like perpetual caregiving roles and recurrent financial strain. As a result, older blacks may be better able to reframe their outlook on life, particularly in old age (34), affecting their psychological and behavioral responses to stress. In an effort to cope with chronic stress, individuals often develop cognitive shifts or changes in how they perceive stress that reduce the stressfulness of exposure (35). black–white differences in the experience of stress are likely the consequence of differences in lifetime exposures to stress that alter perceptions of these exposures and potentially play a protective role for blacks surviving into older adulthood (21).

### Chronic Stress and Mental Health

Chronic stressors are persistent and enduring, often have no easy solution, require ongoing coping, and tend to surface within major social and role domains. As a result, they can elicit a prolonged stress response, leading to psychiatric illnesses such as depression and anxiety disorders (4,36,37). Prior work has suggested that chronic stressors rather than life events were found to be of primary importance in explaining the social distribution of self-rated health (38) and psychopathology (39), making them central to investigating race/ethnic differences in anxiety and depression. Yet, not all individuals exposed to chronic stressors develop anxiety or depressive symptoms and there are documented paradoxical race differences in diagnosed anxiety and depression psychopathology that suggest racial disparities in exposure to chronic stressors are not the sole determinant of subsequent mental health outcomes. There may be meaningful differences in the mental health outcomes for older adults who appraise chronic stressors as less upsetting and who are productively coping to manage chronic stress.

### Distinguishing Anxiety and Depression

Depression and anxiety have substantial comorbidity, partially due to shared genetic and environmental risk factors (40–42)—including family history, gender, medication use, adverse life events, as well as subjective interpretation of one’s conditions as threatening or distressing (43). In fact, a factor analysis of 10 psychiatric conditions found the best fit was three factors, one of which contained major depression, dysthymia (minor depression), and anxiety together (44). However, anxiety and depression are distinctive experiences, anxiety being uniquely characterized by feelings of panic (40). Which condition an individual expresses is said to ultimately be shaped largely by environmental experiences (41). One article found that stressful life events that represent a loss (i.e., death of a spouse, losing a job) are associated with depression while those that represent a danger (i.e., major illness or injury) are associated with anxiety (45). One might especially experience a stressor as a danger, reacting with worry, fear, and anxiety, if they do not have effective coping resources (46). In the face of chronic and ongoing stress, like financial and housing insecurity, older adults may diminish or exhaust their resources in an effort to continuously cope, resulting in long-term anxiety symptoms, which can also lead to the endorsement of depressive symptoms (47). Depressive symptomology is distinct in that it results from feeling sustained hopelessness or prolonged sadness rather than feelings of fear, danger, or panic (48). Depression is not a normal part of aging; however, older adults are at an increased risk for experiencing depression (49). In this study, we examine both anxiety and depressive symptoms because, despite sharing some features, they are different and there are different mechanisms leading to each outcome. Using just one measure in the context of chronic stress might mischaracterize racial differences in the mental health consequences of stress exposure and appraisal.

While we do not examine differences in diagnosed anxiety and depression disorders, examining symptomology in a community-dwelling sample of black and white older adults will help inform gaps in our understanding about black–white patterning of mental health from symptomology to diagnosed disorders. Using symptomology in a survey setting might even be a more comprehensive reflection of black mental health in older community-dwelling adults than diagnosed disorders for two reasons. First, measuring symptomology limits bias due to differential access to health care across respondents that can be introduced by only measuring diagnoses. Self-reports of diagnosed disorders that are often found in nationally representative samples may underestimate and misrepresent health for older black adults who have been historically excluded from the health care system and often distrust and have less access to health care that provides diagnoses (50). Second, symptomology measures generally have more variation than simple yes/no measures of diagnosis (51). As a result, symptomology does not overlook the mental health needs of individuals who do not meet the full diagnostic criteria for anxiety or depressive disorders.

### Current Study

No prior research has independently examined the effects of chronic stress exposure and stress appraisal on black–white differences in mental health. We examine stress exposure and appraisal as interdependent mechanisms that affect anxiety and depressive symptoms among black and white older adults and the domains of chronic stress that are most strongly associated with anxiety and depressive symptoms for both groups. This work aims to interrogate how chronic stressors exert their detrimental effects on mental health and why these effects are stronger for some individuals than others, with specific attention to how appraising chronic stressors as not or less upsetting may be protective against psychopathology. The key contribution of this work is determining if chronic stress appraisal is a mechanism linking stress exposure to black–white differences chronic stress exposure, anxiety, and depressive symptomology.

## Method

Data came from the nationally representative Health and Retirement Study (HRS), an ongoing biennial study of U.S. adults aged 51 and older that began in 1992, with the aim of improving our understanding of the social, economic, environmental, and behavioral factors associated with the health of older adults. In 2006, the HRS included questions about chronic stress using a self-administered questionnaire (SAQ) that was given to a random half-sample of noninstitutionalized respondents. The SAQ had a 90% completion rate, leaving 7168 eligible respondents (52). We excluded 665 respondents who did not identify as white or black, 152 with missingness on depression and anxiety measures, and 332 respondents with missingness on stress measures, resulting in a final analytic sample of 6019 older adults.

### Anxiety

The HRS used 5 items from the Beck Anxiety Inventory (BAI) in the SAQ (53). The BAI has been shown to distinguish symptoms of anxiety from depression and to be valid for use in older populations (18,54). Respondents were asked how often in the past week they felt: *fear of the worst happening*, *nervous*, *hands trembling*, *fear of dying*, and *faint*. Respondents could choose 1 = *never*, 2 = *hardly ever*, 3 = *some of the time*, or 4 = *most of the time* and were told “the best answer is usually the one that comes to your mind first.” Responses to the items were averaged to form an index of anxiety (range = 1–4; α = 0.80) and respondents were considered missing if more than two of the four items had missing values (52).

### Depression

The HRS uses the abbreviated version of the Center for Epidemiologic Studies—Depression scale (CES-D) (55) with eight yes/no items from the original 20-item CES-D, which has been validated for use in older adult populations (56). Respondents were asked if they had experienced the following items in the past week: *felt depressed*, *everything was an effort*, *sleep was restless*, *felt happy*, *felt lonely*, *enjoyed life*, *felt sad*, and *could not get “going.”* Two items (*happy* and *enjoyed life*) were reverse-scored and responses were summed (range = 1–8). Respondents missing three or more depressive symptoms were considered missing on CES-D and not included in this analysis (52).

### Chronic Stress Exposure and Appraisal

Ongoing chronic stress (57,58) was measured by asking respondents to report whether they experienced exposure during the last 12 months or longer to *ongoing health problems (in yourself), physical or emotional problems (in spouse or child), problems with alcohol or drug use in family member, financial strain, housing problems, problems in a close relationship*, and *helping at least one sick/limited/frail family member or friend on a regular basis.* An item assessing ongoing problems in the workplace was excluded from our analysis because more than half of respondents were retired or out of the labor force. For each of the ongoing chronic stressors, respondents could choose: 0 = *no, it didn’t happen*, 1 *= yes, it did happen and it was not upsetting*, 2 *= yes, it did happen and it was somewhat upsetting*, or 3 = *yes, it did happen and very upsetting*.

From this, we created two measures. First, we created a measure of cumulative chronic stress exposure using the sum of the number of chronic stressors respondents reported experiencing (range = 0–7) during the last 12 months or longer based on respondents’ self-reports (yes/no). Next, we created a stress appraisal scale by averaging across responses of how upsetting each stressor was perceived to be (range: 0–3; α = 0.75).

### Sociodemographic Variables

Race is self-reported and respondents are classified as non-Hispanic white or non-Hispanic black. We include sociodemographic and socioeconomic factors that might be related to race differences in stress exposure, appraisal, anxiety, and depression. Age is measured as a continuous variable in years. Gender is dichotomized as male or female. Educational attainment is measured using the number of years of completed schooling. Employment status is categorized as currently employed either full- or part-time, unemployed/not in the labor force, and retired. Total household income is log-transformed and wealth (assets minus debts) is categorized into quartiles because these variables are highly skewed. Marital status is categorized as married/partnered, divorced/separated, widowed, and never married.

### Analytic Strategy

Our first set of models estimate black–white differences in chronic stress exposure, appraisal, anxiety, and depressive symptoms. To describe racial differences in chronic stress exposure, we regress cumulative stress exposure on race, adjusting for age and gender using a Poisson generalized linear model. To describe racial differences in stress appraisal, we use ordinary least squares (OLS) to regress stress appraisal on race, adjusting for age, gender, and stress exposure. For anxiety symptoms, we first use OLS regression models to predict black–white differences in anxiety symptoms adjusting for age and gender. In the second set of models, we estimate black–white differences in patterns of anxiety by stress appraisal (not upsetting, somewhat upsetting, and very upsetting) for each domain of chronic stress exposure (health, financial, housing, relationship, and caregiving) using OLS regression models adjusting for age, gender, and cumulative chronic stress exposure. In a final series of models, we examine the interaction between race and chronic stress exposure on anxiety symptoms—assessing whether stress appraisal moderates this relationship. Here, Model 1 regresses anxiety symptoms on age, gender, education, income, wealth, marital, and employment status. Model 2 adds chronic stress exposure to examine the overall effect of exposure on anxiety symptoms and if doing so alters the magnitude of race differences observed in Model 1. Model 3 includes an interaction between race and stress exposure to assess black–white differences in anxiety across levels of stress exposure. Model 4 adds stress appraisal to determine whether race disparities in stress exposure and anxiety symptoms are conditional on stress appraisal. We repeat this modeling procedure for depressive symptoms using negative binomial regression models because depressive symptoms are an overdispersed count variable. We tested interactions between race and appraisal on anxiety and depressive symptoms in Model 4, but interactions were not significant and thus not included in our final models. All analyses are weighted using the self-administered sampling weights provided by HRS, using the SVY suite of commands in Stata 14.1.

## Results

Table 1 presents weighted sociodemographic characteristics for the full sample and stratified by race. The mean age in the sample was 65.4 (range: 52–101). Women made up about 54% of the sample, 91% were white and the mean level of education was 13.2 years (range: 0–17). The average household income was approximately $68,000 and the mean wealth was approximately $470,000. Nearly 53% of respondents were retired and 69% were married or partnered.

**Table 1.** Weighted Descriptive Statistics for the Full Sample and by Race, Health and Retirement Study, 2006 (*n* = 6019)

	Full Sample (*n* = 6019), %	Whites (*n* = 5219), %	Blacks (*n* = 800), %	*F*
Age (mean [SE]; range 52–101)	65.4 (0.3)	65.6 (0.3)	63.8 (0.5)	12.1**
Female	54.1	53.5	60.0	7.8**
Education (mean [*SE*]; range 0–17)	13.2 (0.0)	13.4 (0.1)	11.8 (0.2)	98.1***
HH income (mean [*SE*]; range 0–16.4)	10.7 (0.0)	10.8 (0.0)	10.0 (0.1)	104.3***
HH wealth				106.2***
First quartile	22.6	19.2	56.1	
Second quartile	25.3	25.2	25.9	
Third quartile	25.6	27.1	11.7	
Fourth quartile	26.5	28.6	6.4	
Employment status				6.6**
Currently employed	37.9	38.3	33.9	
Retired	52.5	52.6	52.0	
Not in the labor force	9.5	9.1	14.1	
Marital status				37.0***
Married	69.2	71.3	48.9	
Divorced/separated	12.0	10.9	22.6	
Widowed	15.33	14.7	21.2	
Never married	3.5	3.1	7.3	
Stress exposure (mean [*SE*]; range 0–7)	2.2 (0.0)	2.1 (0.0)	2.7 (0.1)	60.5***
Stress appraisal (mean [*SE*]; range 0–3)	1.4 (0.0)	1.4 (0.0)	1.5 (0.0)	0.1
Anxiety symptoms (mean [*SE*]; range 1–4)	1.6 (0.0)	1.5 (0.0)	1.7 (0.0)	41.6***
Depressive symptoms (mean [*SE*]; range 0–8)	1.4 (0.0)	1.3 (0.0)	2.0 (0.1)	42.6***

*Note:* HH = household; HH income is logged.

**p < .01, ***p < .001.

When looking at the sample characteristics by race/ethnicity, whites were, on average, older, more educated, and had higher income and wealth than their black counterparts. On average, blacks reported around three chronic stressors (range: 1–7), significantly more than whites who reported around two—and yet blacks had similar average stress appraisal (1.5; range 0–3) as their white peers (1.4). blacks also reported significantly higher mean anxiety and depressive symptomology relative to their white peers. Weighted descriptive statistics of chronic stress exposure and appraisal by race and chronic stress domain are available in Supplementary Table 1.

**Figure 1.**
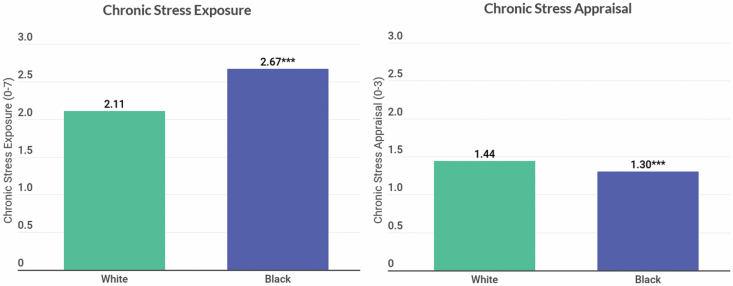
Predicted means showing black–white differences in chronic stress exposure and appraisal (*n* = 6019). *p* Values show significant differences from whites at ***p < .001. Predicted means for chronic stress exposure come from a Poisson regression model adjusted for age and gender. Predicted means for chronic stress appraisal come from an ordinary least squares regression model adjusted for age, gender, and cumulative stress exposure.

**Figure 2.**
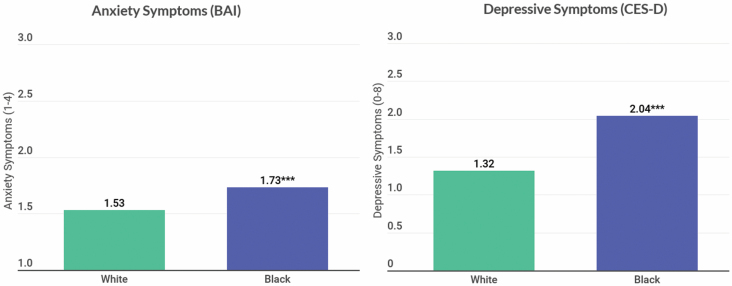
Predicted means showing black–white differences in anxiety and depressive symptoms (*n* = 6019). *p* Values show significant differences from whites at ***p < .001. Predicted means for anxiety symptoms come from an ordinary least squares regression model adjusted for age and gender. Predicted means for depressive symptoms come from a negative binomial regression model adjusting for age and gender. BAI = Beck Anxiety Inventory; CES-D = Center for Epidemiologic Studies—Depression scale.

To further illustrate race differences in chronic stress exposure and appraisal, Figure 1 shows predicted mean black–white differences in chronic stress exposure and appraisal derived from the first set of models described above. Older blacks report greater stress exposure than whites controlling for age or gender. Yet, after adjusting for age, gender, and cumulative chronic stress exposure, blacks report lower average stress appraisal relative to whites. To show black–white differences in anxiety and depressive symptoms, Figure 2 shows older blacks report both more anxiety and depressive symptoms relative to older whites only adjusting for age and gender.

### Chronic Stress Domains

Table 2 presents black–white differences in models predicting anxiety and depressive symptoms by stress appraisal and chronic stress domain, controlling for age, gender, and cumulative chronic stress exposure. Table 2 presents two patterns that set the stage for the multivariable models that follow. First, for both blacks and whites, mean anxiety and depressive symptomology are higher for those who considered their stress exposure *somewhat or very upsetting*, relative to those who consider it *not upsetting*, with the exception of relationship strain. Second, for both whites and blacks, individuals who reported health, financial, and housing strain as *somewhat or very upsetting* reported more average anxiety and depressive symptoms relative to other domains of stress, regardless of how upsetting the stressor was considered to be.

**Table 2.** Black–White Differences in Predicted Mean Anxiety and Depressive Symptoms by Stress Appraisal Within Each Chronic Stress Domain, Health and Retirement Study, 2006 (*n* = 6019)

	Anxiety Symptoms				Depressive Symptoms			
	White		Black		White		Black	
	Mean	CI	Mean	CI	Mean	CI	Mean	CI
Health								
Not upsetting	1.45	1.43–1.48	1.57*	1.51–1.63	1.04	0.96–1.12	1.41*	1.25–1.57
Somewhat upsetting	1.59	1.55–1.63	1.71*	1.64–1.77	1.60	1.49–1.70	2.16*	1.95–2.37
Very upsetting	1.90	1.82–1.98	2.02	1.93–2.11	2.51	2.29–2.72	3.39*	3.00–3.79
Financial								
Not upsetting	1.48	1.44–1.52	1.60*	1.53–1.67	1.11	1.01–1.26	1.62*	2.33–2.90
Somewhat upsetting	1.62	1.57–1.67	1.74	1.66–1.81	1.54	1.38–1.70	1.66	1.44–1.87
Very upsetting	1.86	1.77–1.95	1.98	1.88–2.08	2.52	2.11–2.93	3.02	2.63–3.41
Housing								
Not upsetting	1.56	1.47–1.65	1.67	1.58–1.76	1.30	1.08–1.52	1.52	1.34–1.70
Somewhat upsetting	1.76	1.67–1.85	1.87	1.77–1.97	1.77	1.46–2.08	2.11	1.85–2.36
Very upsetting	1.83	1.65–2.01	1.94	1.74–2.14	2.10	1.52–2.69	3.45*	2.83–4.08
Relationship								
Not upsetting	1.52	1.50–1.55	1.64*	1.57–1.70	1.37	1.29–1.45	1.85*	1.64–2.05
Somewhat upsetting	1.46	1.40–1.51	1.57	1.49–1.65	1.13	0.98–1.29	1.53	1.29–1.76
Very upsetting	1.54	1.43–1.65	1.65	1.52–1.78	0.89	0.72–1.06	1.20	0.95–1.45
Caregiving								
Not upsetting	1.43	1.40–1.46	1.55*	1.49–1.61	0.99	1.36–1.58	1.37	1.18–1.56
Somewhat upsetting	1.53	1.48–1.58	1.65	1.58–1.71	1.18	1.13–1.37	1.63*	1.40–1.86
Very upsetting	1.55	1.45–1.65	1.67	1.55–1.79	1.58	1.60–1.96	2.18	1.71–2.64

*Note:* Models adjusted for age, gender, and cumulative stress exposure.

*Different from whites at *p* < .05.

### Anxiety Symptoms

**Figure 3.**
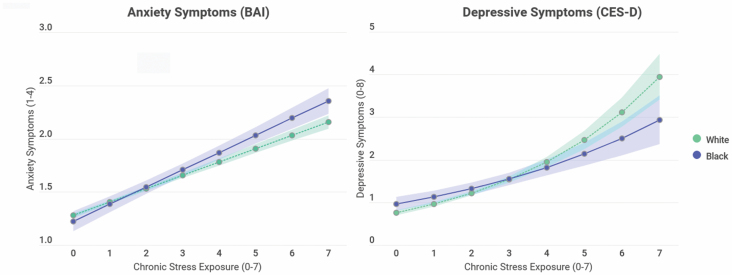
Predicted means showing black–white differences in anxiety and depressive symptoms with chronic stress exposure (*n* = 6019). Predicted means for anxiety come from Table 3, Model 3 adjusted for age, gender, education, income, wealth, marital, and employment status. Predicted means for depressive symptoms come from Table 4, Model 3 adjusted for age, gender, education, income, wealth, marital, and employment status. BAI = Beck Anxiety Inventory; CES-D = Center for Epidemiologic Studies—Depression scale.

Table 3 presents results from OLS models predicting anxiety symptoms by race, stress exposure, and stress appraisal. Model 1 establishes race differences in anxiety symptomology, with older blacks reporting more anxiety symptoms than whites after adjusting for age, gender, education, income, wealth, marital, and employment status (Model 1: β = 0.07, *SE* = 0.03; *p* < .05). Model 2 adds chronic stress exposure showing anxiety symptoms increase with the number of chronic stress exposures (Model 2: β = 0.13, *SE* = 0.01; *p* < .001). Model 2 also demonstrates that chronic stress exposure accounts for race differences in anxiety symptoms (Model 2: β = 0.04, *SE* = 0.03; *p* > .05). The interaction between race and stress exposure in Model 3 is significant, suggesting that the effect of stress exposure on anxiety differs by race (Model 3: β = 0.04, *SE* = 0.01; *p* < .01). Figure 3 graphs the interaction from Model 3 showing that at lower levels of exposure, blacks report similar anxiety symptoms as whites, but at higher levels of exposure blacks are more likely to report more symptoms than whites. Model 4 adds stress appraisal, which is a significant independent predictor of anxiety symptoms (Model 4: β = 0.10, *SE* = 0.01; *p* < .001). Including stress appraisal slightly increases the interaction coefficient between race and stress exposure (Model 4: β = 0.05, *SE* = 0.01; *p* < .001), suggesting the relationship between race, chronic stress exposure, and anxiety is—in part—mediated by chronic stress appraisal.

**Table 3.** OLS Regression Models Predicting Anxiety Symptoms, Health and Retirement Study, 2006 (*n* = 6019)

	Model 1		Model 2 (+chronic stress exposure)		Model 3 (+stress exposure interaction)		Model 4 (+stress appraisal)	
Independent Variables	β	*SE*	β	*SE*	β	*SE*	β	*SE*
black (ref = White)	0.07	0.03*	0.04	0.03	−0.06	0.05	−0.07	0.05
Age	0.00	0.00***	0.00	0.00	0.00	0.00	0.00	0.00
Female	0.05	0.01***	0.03	0.02*	0.03	0.02*	0.02	0.02
Education	−0.02	0.00***	−0.02	0.00***	−0.02	0.00***	−0.02	0.00***
HH income	−0.03	0.01*	−0.01	0.01	−0.01	0.01	−0.01	0.01
HH wealth (ref = first quartile)								
Second quartile	−0.12	0.03***	−0.04	0.02	−0.04	0.02	−0.03	0.02
Third quartile	−0.17	0.03***	−0.06	0.02*	−0.06	0.02*	−0.07	0.02*
Fourth quartile	−0.19	0.03***	−0.07	0.03*	−0.07	0.03*	−0.07	0.03*
Employment status (ref = employed)							0.00	
Retired	0.13	0.04**	0.10	0.03**	0.09	0.03**	0.09	0.03**
Not in labor force	0.09	0.02***	0.06	0.02**	0.06	0.02**	0.06	0.02*
Marital status (ref = married)								
Divorced/separated	−0.02	0.03	0.00	0.03	0.00	0.03	−0.02	0.03
Widowed	0.02	0.02	0.04	0.02	0.05	0.02	0.03	0.02
Never married	0.00	0.06	0.06	0.06	0.06	0.06	0.06	0.05
Chronic stress exposure (0–7)			0.13	0.01***	0.13	0.01***	0.10	0.01***
black × Exposure					0.04	0.01**	0.05	0.01***
Stress appraisal (0–3)							0.10	0.01***
Intercept	2.37	0.14***	1.69	0.11***	1.71	0.11***	1.66	0.11***

*Note:* HH = household; OLS = ordinary least squares.

*p < .05, **p < .01, ***p < .001.

### Depressive Symptoms

Next, we examined race, stress exposure, and appraisal differences in depressive symptoms using negative binomial regression, presenting incidence rate ratios (IRRs; Table 4). Model 1 shows that blacks report higher levels of depressive symptoms compared to whites (Model 1: β = 1.05, *SE* = 0.06; *p* < .001) after adjusting for our demographic and SES measures. Chronic stress exposure significantly and positively predicts depressive symptoms in Model 2 (IRR = 1.25, *SE* = 0.02, *p* < .001) and attenuates race differences in depressive symptomology (Model 2: IRR = 1.02, *SE* = 0.06, *p* > .05). Similar to our findings on anxiety symptoms, the interaction between race and stress exposure is significant in fully adjusted models (Model 3: IRR = 0.93, *SE* = 0.02, *p* < .01), resulting in differential depressive symptomology for blacks and whites. Figure 3 graphs the interaction from Model 3 showing that at lower levels of exposure, blacks are more likely to report depressive symptoms than whites, but at higher levels of exposure blacks are less likely to report depressive symptoms. This black–white crossover occurs at approximately three reported chronic stressors, demonstrating a paradoxical relationship for depressive symptoms among blacks, with fewer depressive symptoms at higher levels of stress exposure relative to whites. In Model 4, stress appraisal is a significant independent predictor of depressive symptoms (Model 4: β = 1.46, *SE* = 0.05; *p* < .001) and attenuates the interaction between race and stress exposure (Model 4: β = 0.96, *SE* = 0.03; *p* > .05). Stress appraisal fully explains the effect of black–white differences in chronic stress exposure on depressive symptoms.

**Table 4.** Negative Binomial Regression Models Predicting Depressive Symptoms, Health and Retirement Study, 2006 (*n* = 6019)

	Model 1		Model 2 (+chronic stress exposure)		Model 3 (+stress exposure interaction)		Model 4 (+stress appraisal)	
Independent Variables	IRR	*SE*	IRR	*SE*	IRR	*SE*	IRR	*SE*
Black (ref = white)	1.05	0.06***	1.02	0.06	1.27	0.12*	1.21	0.12*
Age	0.99	0.00***	1.00	0.00	1.00	0.00	1.00	0.00
Female	1.07	0.04	1.02	0.04	1.02	0.04	0.99	0.04
Education	0.95	0.01***	0.94	0.01***	0.94	0.01***	0.94	0.01***
HH income	0.92	0.03**	0.95	0.02*	0.95	0.02*	0.95	0.02*
HH wealth (ref = first quartile)								
Second quartile	0.80	0.05***	0.92	0.06	0.91	0.06	0.93	0.06
Third quartile	0.76	0.05***	0.92	0.05	0.92	0.05	0.92	0.05
Fourth quartile	0.66	0.05***	0.81	0.05**	0.82	0.05**	0.81	0.05***
Employment status (ref = employed)								
Retired	1.52	0.14***	1.44	0.13***	1.44	0.13***	1.42	0.13***
Not in labor force	1.27	0.07***	1.18	0.07*	1.18	0.07**	1.15	0.07*
Marital status (ref = married)								
Divorced/Separated	1.33	0.08***	1.41	0.08***	1.41	0.08***	1.35	0.08***
Widowed	1.43	0.07***	1.50	0.08***	1.50	0.08***	1.43	0.07***
Never married	1.21	0.16	1.45	0.20**	1.45	0.20**	1.42	0.18**
Chronic stress exposure (0–7)			1.25	0.02***	1.27	0.02***	1.15	0.02***
black × Exposure					0.93	0.02**	0.96	0.03
Stress appraisal (0–3)							1.46	0.05***
Intercept	11.59	4.03***	2.86	0.88**	2.70	0.83**	2.02	0.61*

*Note:* HH = household; IRR = incidence rate ratio.

*p < .05, **p < .01, ***p < .001.

## Discussion

This study is the first to examine black–white differences in chronic stress exposure and appraisal as principal and interdependent mechanisms contributing to mental health disparities in a diverse nationally representative sample of older adults. Stress measures at the population level have not captured race differences in stress appraisal, or the individual variability in how one evaluates the stressfulness of any given stress exposure, and thus overlooks a pathway that may drive paradoxical race differences in mental health outcomes. This article is innovative in that we demonstrate that aspects of the stress experience, beyond stress exposure, are often not measured in population surveys but are essential to understanding population differences in mental health. Stress exposure measures were developed primarily to assess stress among normative middle-aged whites (59) and without incorporating subjective aspects of the stress experience. We find stress appraisal is a subjective but tandem mechanism through which race differences in stress exposure operate to both reduce or exacerbate the detrimental effects of stress on mental health. This study also implicates the domains of chronic stress exposure (health, financial, housing, relationship, and caregiving strain) that are most consequential for mental health outcomes for blacks and whites.

First, it is important to note that there is no evidence of a black–white mental health paradox in anxiety and depressive symptoms among our sample of older black and white adults until we adjust for chronic stress exposure. Older blacks in our sample report more chronic stress exposure and more anxiety and depressive symptoms relative to older whites. Once we take into account chronic stress exposure, blacks and whites report similar levels of anxiety and depressive symptomology. This is consistent with findings showing higher levels of psychological distress among blacks relative to whites and is in line with prior studies that show similar or lower rates of diagnosed or depressive and anxiety symptomology among blacks despite higher levels of stress exposure (12). Importantly, despite being exposed to more chronic stressors and reporting more anxiety and depressive symptoms, older blacks in this sample appraise exposure to chronic stressors as less upsetting than older whites.

In examining chronic stress exposure and appraisal by domain, black and white older adults who report exposure to health, financial, or housing strain report more anxiety and depressive symptoms than those who reported exposure to any other type of chronic stressor, especially if they consider these exposures very upsetting. Health strain results in significantly more anxiety and depressive for blacks, despite the general belief and theories of health behavior that imply that blacks care less about their physical health relative to whites (16). While this article is not directly measuring anxiety and depression as a result of the coronavirus pandemic, understanding the impact of chronic health problems on the mental health of older adults represents an important frontier. Older Americans are currently facing a plethora of uncertainties with respect to their health, worries which are exacerbated amid some of the isolation due to social distancing policies of the pandemic. From this framework, these findings may help us anticipate the short- and long-term effects of living through a pandemic, demanding an immediate focus on prevention and direct intervention to address the impact on individual- and population-level mental health among older adults.

We also establish an important link between financial and housing strain in late life and mental health. These are severe and often unrelenting stressors patterned by social and structural disadvantage over the life course, capturing the major hardships in late life for blacks and whites (8). Several studies have documented broad psychological distress or depression among older adults who face financial hardship (60,61). Older adults who have difficulty paying bills have been shown to delay or forego taking medications due to cost, which can have a devastating impact on mental health symptoms (62,63) in addition to their physical health (50). These findings suggest that experiencing financial or housing insecurity in older adulthood poses a threat to the mental health of older adults. Exposure to financial and housing strain is not equally distributed in our sample. Sixty percent of older blacks report ongoing financial strain compared to 37% of older whites and 23% of blacks relative to 8% of whites report exposure to housing strain. Occupying social positions that disproportionally expose blacks to financial and housing strain may be one mechanism driving race differences in anxiety and depressive symptoms in older adulthood. Amid the additional layer of economic uncertainty faced by many during the coronavirus pandemic (64), alleviating financial insecurity and ensuring adequate and stable housing for older adults are important strategies for lowering anxiety and depressive symptoms in late life.

In examining chronic stress exposure, consistent with prior research (5,21), we found exposure to be an important predictor of both anxiety and depressive symptoms for older adults. Yet, interactions between race and stress exposure show that exposure to chronic stressors differentially predicts anxiety and depressive symptoms for blacks and whites, even after adjusting for potential differentials in protective resources such as SES and marital status. blacks who report exposure to four or more chronic stressors report significantly greater anxiety symptoms than whites with similar stress levels. This finding is important because older blacks are twice as likely to report four or more stressors than older whites; 30% of older blacks compared to only 15% of white adults report exposure to four or more chronic stressors. In line with the stress process model, our older black sample reports more anxiety symptoms relative to whites because a larger share of older blacks report exposure to four or more chronic stressors and specifically to chronic stressors that have detrimental mental health effects like health, financial, and housing strain.

Conversely, for depressive symptoms, interactions between race and cumulative stress exposure show blacks reporting greater exposure to chronic stressors report fewer symptoms relative to whites with similar cumulative stress exposure. This finding aligns with studies examining the black–white mental health paradox which has primarily shown the black mental health advantage to be most evident for major depression (12,14). These studies have generally found that despite greater stress exposure, material hardship, and worse physical health, black Americans tend to experience similar or relatively lower rates of depression relative to whites (15,16). Our study concurrently suggests that black older adults report fewer depressive symptoms relative to older whites but more anxiety symptoms at higher levels of chronic stress exposure. Thus, stress exposure does not predict anxiety and depressive symptoms uniformly for older whites and blacks, though the literature often treats anxiety and depressive symptoms as exchangeable outcomes for these groups. The distinction may lie in the hallmark features of depression—hopelessness and prolonged sadness—whereas anxiety is generally thought to be more immediate and fear-based (40). Prior findings also suggest that blacks are more likely than whites to somaticize distress (65). It may be that stress exposure translates into different mental health symptoms for whites and blacks, or racial differences in exposures to specific stressors result in differential endorsement in symptomology. For example, domains that are the most patterned by race at the oldest ages (i.e., financial or housing) may result in the endorsement of more physiological responses in blacks and three of the five anxiety symptoms in the BAI are physiological compared to only two of the eight depressive symptoms. In any instance, stress exposure does not translate uniformly across groups and, as a result, may not reveal the entire stress experience of older blacks. Future research focused on mechanisms by which stress exposure relates to mental health outcomes should disentangle differences in exposure as they relate to anxiety and depressive symptoms, general distress, and psychiatric diagnoses because these outcomes have inconsistent black–white patterning.

Stress appraisal, an understudied yet critical feature of the stress process, offers an important explanation for race differences in mental health. Older blacks in this sample appraise their stress as less upsetting compared to whites after controlling for their overall chronic stress exposure (21). Stress appraisal independently predicts anxiety and depressive symptoms and it helps explain how black–white differences in chronic stress exposure affect mental health symptomology. Stress appraisal partially mediates the interaction between race and stress exposure on anxiety symptoms and fully mediates the interaction between race and stress exposure on depressive symptoms. Similar to the black–white mental health paradox in diagnosed disorders, we find a paradoxical relationship with race, stress exposure, and depression showing that blacks report lower levels of depressive symptoms with greater stress exposure relative to whites. This paradoxical finding, or the interaction between race and stress exposure on depressive symptoms, is fully explained by stress appraisal suggesting paradoxical race differences in diagnosed disorders may be explained by the subjective components of the stress process. That is, whether or not a stressor is considered upsetting in the first place may be one mechanism through which black older adults reduce the detrimental effects of disproportionate stress exposure on mental health, making a strong case for measuring appraisal in connection to stress exposure. Demonstrating the role of stress appraisal on mental health symptomology has broader implications for the black–white mental health paradox in psychiatric disorders, especially for diagnosed depression. Stress appraisal is an overlooked pathway by which blacks may simultaneously report more exposure to stressors relative to whites but also report better mental health.

Scholars increasingly recognize that the pathways to mental health unfold in different ways for black and white Americans. One hypothesis that has emerged in the literature to explain the black–white paradox in mental health is that these groups differ in how they respond to stress. For example, black older adults have access to racially salient positive resources (i.e., religiosity, social support, and racial identity) that may buffer the effects of stress on health. Although recent studies suggest that neither religious involvement (28), family relationships (29), nor relationships of choice (29) appear to serve as positive coping mechanisms to explain the black–white paradox in mental health, it is highly plausible that given a prolonged history of marginalization, older blacks have developed other coping mechanisms that may account for their mental resilience. Older blacks, who appraise chronic stress as less upsetting relative to whites across every domain (21), may have found adaptive means or have habituated to greater stress exposure by reframing stress or developing cognitive shifts to reduce the stressfulness of exposure (35,66). The black older adults in our sample came of age during Jim Crow, desegregation, and the Civil Rights Era and thus may perceive the chronic stressors that are measured here as less stressful because they have lived through very overt periods of racism and discrimination. It may also be that older blacks who are able to effectively cope with a chronic stressor report perceiving it as less severe over time even if it was a more intense stressor when the experience initiated. Importantly, these hypotheses engage race- and age-specific stress and coping mechanisms that highlight the distinct stress experience for older blacks when trying to better understand black–white differences in mental health, a point that is relevant for all future black–white paradox work.

### Study Limitations

This study has a few limitations in the way we measure and conceptualize stress exposure and appraisal. First, one of the problems with studying appraisals is that their location in the stress process is unclear. While we use a measure of appraisal that has been utilized in other studies (57), the retrospective timing in which the questions are asked requires respondents to report the stressfulness of chronic situations, even if it is not affecting them at the moment. Individuals may be reporting stress exposure during the past 12 months but at the point of the interview may be feeling less bothered by the stressor. Rather than older adults at the moment appraising a situation as less stressful, they may report it as less stressful because it did not ultimately affect their mental health, leaving us unable to rule out reverse causality. However, there is no reason to think this would be more common among blacks than whites and likely would not change the race differences we find. Additionally, stronger selective mortality among blacks than whites may make the blacks in this sample a select group of individuals who experienced less chronic stress, coped well, or who responded better to stressors and, as a result, were more likely to survive to old age. Importantly, we are measuring chronic stress cross-sectionally when the relationship between race/ethnicity, stress exposure and appraisal, and mental health may vary over time. This question about ongoing chronic stress in the HRS was only asked in 2010 and then asked again to the same half sample of older adults in 2014. We did look at the change in that 4-year period; however, not surprisingly, chronic stress is fairly stable in that window, speaking to the persistence of chronic strains in the lives of older adults in our sample. Thus, it made sense for us to only consider one wave of data, especially when we considered issues of attrition between waves. Finally, in measuring the “stress universe,” it would be appropriate to note the importance of including a wider array of race-based or related stressors (e.g., vicarious discrimination, incarceration, and intersectional stressors) in future research on race/ethnic differences in the stress process (67,68).

## Conclusions

The black–white mental health paradox—or the phenomenon of blacks reporting similar or better mental health compared to whites despite greater exposure to stressors—is a key empirical finding that suggests that factors other than stress exposure contribute to race differences in psychological well-being. Appraisal processes are not routinely measured at the population level, leaving gaps in our understanding about the mental health impacts of stress, one potential explanation for the black–white mental health paradox. The central finding of this article is that the degree to which a person perceives a stress exposure as a threat is crucial in determining the mental health consequences of stress. Measuring both chronic stress exposure and stress appraisal depicts the interdependent effects of the stress experience on the mental health of older adults.

The impact of chronic stress on mental health is aptly demonstrated by new Census Bureau data from the Household Pulse Survey, showing that anxiety and depressive symptoms have more than tripled compared to symptoms reported before the coronavirus pandemic (64,69). Older Americans are currently facing a plethora of uncertainties with respect to their health, worries which are exacerbated amid some of the isolation due to social distancing policies. The coronavirus pandemic places an undue burden on older black adults who are at disproportionate risk of mortality (70) coupled with the fact that black Americans face a higher risk of police-involved death (33). Police-involved shootings (71) and chronic community violence (72) have a collective toll on black well-being and increase the risk of anxiety and depressive symptoms. The intersection of these chronic stressors will undoubtedly have consequences for the mental health and well-being of older black adults and necessitate an immediate focus on prevention and direct intervention to address their impact on individual- and population-level mental health.

Using a multidisciplinary framework that builds on the stress process model (48,73,74), we also place a specific emphasis on the disproportionate chronic stress exposure older black adults face to health, housing, and financial strain. Practice and policy implications of the study call for connecting black older adults facing health, financial, and housing adversity to services that minimize mental-health-related repercussions. Alternatively, establishing financial and housing security as fundamental rights for black older people lessens exposure to chronic stressors that have detrimental mental health consequences. Yet, it is also historically inaccurate to reduce the experience of black people to the vast amounts of stress and suffering inflicted upon them. black Americans have been and continue to cope, fight, love, have families, and live despite the adversities they face. This article acknowledges their unequal burden of exposure to adversity, but not without also acknowledging the resourcefulness, resilience, agency, and effort black Americans have used and are using to survive into older adulthood. A story without both is not complete, and black Americans should not only be defined by their stress or trauma. Future research should conceptualize and measure aspects of the stress experience, like stress appraisals, that may be distinct for minority populations, disentangling how these unique aspects affect their physical and mental health.

## Supplementary Material

igaa040_suppl_Supplementary_Table_1
