# Supplementary material for: Measuring More Than Exposure: Does Stress Appraisal Matter for Black–White Differences in Anxiety and Depressive Symptoms Among Older Adults?
Source: Innov Aging. 2020 Sep 7;4(5):igaa040. doi: 10.1093/geroni/igaa040 (PMC7580160; doi:10.1093/geroni/igaa040)
Supplement: igaa040_suppl_Supplementary_Table_1 [file igaa040_suppl_supplementary_table_1.docx]

Online Supplementary Material for *Innovation in Aging:*

Brown, LL, Abrahams, LR, Mitchell, U, Ailshire, JA. Measuring more than exposure: Does stress appraisal matter for Black-White differences in anxiety and depressive symptoms among older adults?. *Innov Aging. 2020.*

| Supplement A. Weighted descriptive statistics of chronic stress exposure and appraisal by race and chronic stress domain, Health and Retirement Study, 2006 (n=6,019) | | | | |
| --- | --- | --- | --- | --- |
|  | Full Sample | Whites | Blacks |  |
|  | (n=6,019) | (n=5,219) | (n=800) |  |
|  | % | % | % | F |
| **Cumulative Stress Exposure [mean(SE); range 0-7]** | 2.2(0.0) | 2.1(0.0) | 2.7(0.1) | 60.5*** |
| Health (% exposed) | 61.3 | 60.6 | 67.7 |  |
| Financial (% exposed) | 39.1 | 37.0 | 59.7 |  |
| Housing (% exposed) | 9.6 | 8.2 | 23.2 |  |
| Relationship (% exposed) | 48.7 | 48.2 | 53.9 |  |
| Caregiving (% exposed) | 35.9 | 35.5 | 39.6 |  |
| **Stress Appraisal Scale [mean(SE); range 0-3]** | 1.4(0.0) | 1.4(0.0) | 1.5(0.0) | 0.1 |
| Health [mean(SE); range 0-3] | 1.1(0.0) | 1.0(0.0) | 1.2(0.0) |  |
| Financial [mean(SE); range 0-3] | 0.7(0.0) | 0.6(0.0) | 1.0(0.0) |  |
| Housing [mean(SE); range 0-3] | 0.2(0.0) | 0.1(0.0) | 0.4(0.0) |  |
| Relationship [mean(SE); range 0-3] | 0.7(0.0) | 0.7(0.0) | 0.8(0.0) |  |
| Caregiving [mean(SE); range 0-3] | 0.5(0.0) | 0.5(0.0) | 0.6(0.0) |  |
